# 

**Published:** 1888-04

**Authors:** 


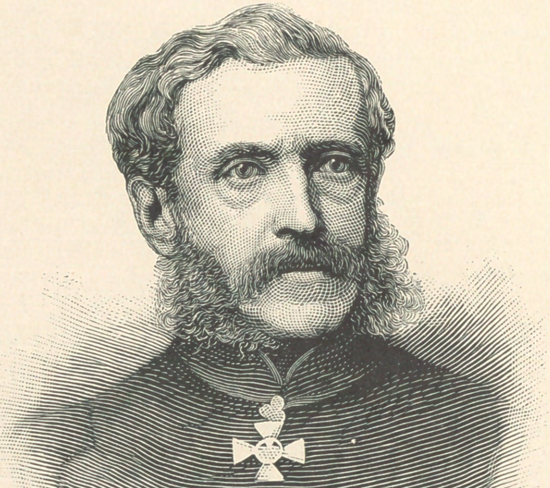

B. LANGENBECK, M. D.

### BOOKS RECEIVED.

Mortality and Vital Statistics, Part II, with Plates and Diagrams. Volume XII, of the Tenth Census of the United States.

Ledger of Monthly Balances and Index of Accounts. A companion to the Medical World Visiting List.

On the Wasting Diseases of Infants and Children. By Eustace Smith, M. D.

The Refraction of the Eye. A Manual for Students. By Gustave Hartridge, F. R. C. S.

Nasal Polypus, with Neuralgia, Hay-Fever, and Asthma, it relation to Ethmoiditis. By Edward Woakes, M. D., London.

A Synopsis of the Physiological Action of Medicines. By Louis Starr, M. D.

Chemical Analysis of Healthy and Diseased Urine. By T. C. Van Nüys.

The Rules of Aseptic and Antiseptic Surgery, By Arpad G. Gerster, M. D.

Transactions of the College of Physicians of Philadelphia.

A Pratical Treatise on Diseases of the Skin. By John V. Shoemaker, A. M. M. D.

Diseases of the Skin. A Manual for Practitioners and Students. By W. Allan Jamieson, M. D., F. R. C. P., Ed.

A Practical Treatise on Medical and Surgical Electricity. By Beard & Rockwell. Revised edition.

Diseases of the Heart and Circulation. By John M. Keating, M.D., and William A. Edwards, M.D.

A Manual of Physiology. A Text Book for Students. By George F. Yeo, M. D., F. R. C. S.

Obstetric Synopsis. By John S. Stewart, M. D.

Irregularities of the Teeth and their Treatment. By Eugene S. Talbot, M. D., D.D. S.

The Treatment of Hæmorrhoids by Injection of Carbolic Acid and other Substances. By Silas T. Young, M. D.

Questions and Answers on the Essentials of Physiology. By H. A. Hare, B. Sc., M. D.

### ANNOUNCEMENTS.

### CONGRESS OF AMERICAN PHYSICIANS AND SURGEONS.

Premliminary programme of the Congress to be held in Washington, D, C., on the evenings of September 18, 19 and 20, 1888.

President: John S. Billings, M.D., U. S. A.

Vice-Presidents, ex-offico: President of the American Surgical Asssociation, D. Hayes Agnew, M. D., Philadelphia, Pa. President of the American Association of Genito-Urinary Surgeons, Edward L. Keyes, M.D., New York City. President of the American Laryngological Association, Rufus P. Lincoln, M.D., New York City. President of the American Climatological Association, Alfred L. Loomis, M.D., New York City. President of the Association of American Physicians, William H. Draper, M.D., New York City. President of the American Otological Society, Jonathan S. Prout, M.D., Brooklyn, N. Y. President of the American Ophthalmological Society, William F. Norris, M.D., Philadelphia, Pa. President of the American Neurological Association, James J. Putnam, M.D., Boston, Mass. President of the American Dermatological Association, I. E. Atkinson, M. D., Baltimore, Md. President of the American Physiological Society, Henry P. Bowditch, M.D., Boston, Mass. President of the American Orthopedic Association, Newton M. Shaffer, M.D., New York City.

Chairman of the Executive Committee, William Pepper, M.D., Philadelphia, Pa. Treasurer, D. B. St. John Roosa, M.D., New York City. Secretary, William H. Carmalt, M.D., New Haven, Conn.

Subjects for Report and Discussion.—Tuesday evening, September 18, "Intestinal Obstruction in its Medical and Surgical Relations." Papers will be read by Dr. Reginald H. Fitz, of Boston, Professor of Pathological Anatomy in Harvard University, and Dr. Nicholas Senn, of Milwaukee, Professor of Surgery in the College of Physicians and Surgeons, in Chicago, Ill., followed by a discussion.

Wednesday evening, September, 19, "Cerebral Localization in its Practical Relations." Papers will be read by Dr. Charles K. Mills, of Philadelphia, Professor of Diseases of the Mind and Nervous System in the Philadelphia Polyclinic and College for Graduates in Medicine, and Dr. Roswell Park, Professor of Surgery in the Buffalo Medical College, followed by a discussion.

Thursday evening, September 20, Address by the President, John S. Billings, M.D., U. S. Army, to be followed by a general reception in the United States Army Museum building.

---

Gari Prize on Blennorrhagia.—The Royal Academy of Medicine and Surgery of Barcelona has announced as the subject of the Gari Prize (of $300) for 1889, "The Pathogenesis of Blennorrhagia; clinical forms of the disease; cases of long duration and recurring cases; concomitant and consecutive affections; prophylactic and curative treatment." Demonstrations and reports of cases should accompany manuscript. These should be sent, written in Spanish, French, or Italian, to the Academy (*Banos Nuevos*, No. 9, Barcelona, Spain) before June 30, 1889.

Volume LVI.] APRIL, 1888. [Number 4.

### The CHICAGO MEDICAL JOURNAL AND EXAMINER

EDITOR:
S. J. JONES, M.D., LL.D

COLLABORATORS:

CHICAGO:

Professor E. ANDREWS, M.D., LL.D.

Professor J. BARTLETT, M.D.

Professor W. T. BELFIELD, M.D.

Professor F. BILLINGS, M.D.

Professor W. H. BYFORD, A.M., M.D.

Professor L. CURTIS, A.M., M.D.

Professor N. S. DAVIS, Jr., A.M., M.D.

Professor O. C. DeWOLF, A.M., M.D.

Professor E. C. DUDLEY, A.B., M.D.

Professor C. FENGER, M.D.

Professor J. N. HYDE, A.M., M.D.

Professor E. F. INGALS, A.M., M.D.

Professor F. S. JOHNSON, A.M., M.D.

Professor H. A. JOHNSON, M.D., LL.D.

Professor C. W. PURDY, M.D.

Professor C. G. SMITH, A.M., M.D.

Professor E. WING, A.M., M.D

MILWAUKEE, WIS.

Professor N. SENN, M.D., Ph. D.

WASHINGTON, D. C.:

S. O. RICHEY, M.D.

UNITED STATES ARMY:
SURGEON D. L. HUNTINGTON, M.D.

UNITED STATES NAVY
SURGEON GENERAL J. M. BROWNE, M.D.
MEDICAL DIRECTOR A. L. GIHON, A.M., M.D.

MARINE HOSPITAL SERVICE:
SURGEON S. T. ARMSTRONG, M.D., Ph.D.

Rand, McNally & Company, Printers.

1888.

